# Ubiquitin recognition of BAP1: understanding its enzymatic function

**DOI:** 10.1042/BSR20171099

**Published:** 2017-10-27

**Authors:** Pranita Hanpude, Sushmita Bhattacharya, Abhishek Kumar Singh, Tushar Kanti Maiti

**Affiliations:** 1 Laboratory of Functional Proteomics, Regional Centre for Biotechnology, NCR Biotech Science Cluster, 3rd Milestone Gurgaon-Faridabad Expressway, Faridabad 121001, Haryana, India; 2 Department of Biotechnology, Manipal University, Karnataka 576104, India

**Keywords:** Deubiquitinating enzyme, Enzyme, Interaction, Mutation, Ubiquitin

## Abstract

BRCA1-associated protein 1 (BAP1) is a nuclear localizing UCH, having tumor suppressor activity and is widely involved in many crucial cellular processes. BAP1 has garnered attention for its links with cancer, however, the molecular mechanism in the regulation of cancer by BAP1 has not been established. Amongst the four UCHs, only BAP1 and UCHL5 are able to hydrolyze small and large ubiquitin adducts but UCHL5 hydrolyzes only when it is present in the PA700 complex of the proteasome. The ability of BAP1 to cleave large ubiquitin derivatives is because of its relatively longer active-site crossover loop than other UCHs. The mechanism of ubiquitin recognition has not been studied for BAP1. The comparative enzymatic analysis of ubiquitin C-terminal hydrolase L1 (UCHL1), ubiquitin C-terminal hydrolase L3 (UCHL3), ubiquitin C-terminal hydrolase L5 (UCHL5N), and BAP1N has confirmed that enzymatically BAP1 is similar to UCHL5, which corroborates with the bioinformatics analysis done earlier. We have undertaken extensive mutational approaches to gain mechanistic insight into BAP1–ubiquitin interaction. Based on the homology-modeled BAP1 structure, we have identified a few BAP1 residues which possibly play a crucial role in ubiquitin interaction of which a few mutations have been identified in many cancers. Our comparative thermodynamic analysis reveals that BAP1–ubiquitin interaction is majorly driven by entropy factor which is unique amongst UCHs. Our study sheds light on BAP1 interaction with ubiquitin, which will be useful in understanding its enzymatic function.

## Introduction

BRCA1-associated protein 1 (BAP1) is a nuclear localizing deubiquitinating enzyme and a tumor suppressor protein, which was initially identified as an interactor of BRCA1 [1]. BAP1 is involved in numerous cellular processes including stem cell pluripotency, DNA damage response and replication, cell cycle progression, histone modification, gluconeogenesis, and myeloid transformation [2–5]. BAP1 regulates expression of various target genes by deubiquitinating transcription-related proteins such as FOXK2, HCF-1, OGT, LSD2, MBD-6, MBD-5, ASXL1, and ASXL2 [6–10]. BAP1 disrupts the BRCA1/BARD1 heterodimer, which acts as an E3 ligase but cannot reverse its auto-ubiquitination [11–13]. The tumor suppressor function of BAP1 is dependent both on its nuclear localization and deubiquitinase activity [12]. In our previous study, we have reported that cancer-associated BAP1 catalytic N-terminal domain mutations abrogate its cellular functions due to the formation of β-amyloid aggregates [14]. During the last few years, BAP1 has gained a clinical interest as a critical tumor suppressor as its loss leads to induction of many cancers [15] including lung cancer, breast cancer, uveal melanoma, meningioma, pleural mesothelioma, and renal cell carcinoma [6,16–28].

BAP1 is a cysteine protease with a conserved catalytic UCH domain sharing 44, 26, and 23% of sequence identity with ubiquitin C-terminal hydrolase L5 (UCHL5), ubiquitin C-terminal hydrolase L3 (UCHL3), and ubiquitin C-terminal hydrolase L1 (UCHL1), respectively. On the basis of sequence similarity, it is predicted that BAP1 has a UCHL5-like domain (ULD) at C-terminal that is potentially important in regulating its enzymatic activity by interacting with its binding partners. It is likely that ULD of BAP1 is structurally and functionally similar to that of UCHL5. Recent studies reveal that mechanism of H2A deubiquitination by BAP1–ASXL1 in PR–DUB complex is similar to UCHL5–RPN13 complex [29,30] where DEUBiquitinase ADaptor domain (DEUBAD) [31] of ASXL1 interacts with ULD of BAP1 and increases the affinity of BAP1 for ubiquitin [32,33]. In BAP1, there are ∼350 amino acids inserted between its UCH catalytic domain and C-terminal ULD domain which play an important role in regulating its functions by interacting with proteins like host cell factor 1 (HCF1). Our previous study has shown that BAP1N exhibits a higher catalytic activity than full-length BAP1, which suggests that BAP1 self-regulates enzymatic activity via its C-terminal domain [14].

Amongst four UCHs, physiological substrates for UCHL1 and UCHL3 are not known but they can hydrolyze C-terminal peptide tail of small ubiquitin derivatives [34]. They fail to process large ubiquitin chains because of their short active site crossover loops. However, BAP1 and UCHL5 possess relatively long crossover loops, which allow them to disassemble polyubiquitin chains efficiently in the presence of appropriate binding partners [33,35]. It has been reported that BAP1N not only can cleave the isopeptide and peptide-linked ubiquitin extensions from structured proteins but also has specificity for cleavage of K48 polyubiquitin chains [36]. The overall folding pattern of BAP1N is similar to UCHs. However, mechanistic insights into BAP1–ubiquitin interaction remain enigmatic.

In the present work, we have shown how BAP1 interacts with the ubiquitin by studying the enzyme kinetics and thermodynamics of BAP1N–ubiquitin interaction. We have predicted a few residues in BAP1N that might play an important role in BAP1–ubiquitin binding based on the ubiquitin-binding interface of analogous UCHs. It is interesting to note that some of the putative ubiquitin interacting residues are found to be mutated in many cancers as observed in the **C**atalogue **O**f **S**omatic **M**utations **I**n **C**ancer (COSMIC) database. To gain a further mechanistic insight into UCHs function, the comparative thermodynamic analysis of ubiquitin binding to BAP1N and other UCH members has been performed. The thermodynamic studies of UCH members and ubiquitin disclose a differential nature of substrate interactions in UCH members.

## Materials and methods

### Cloning, expression, and purification

Flag-HA tagged full-length BAP1, UCHL3, and UCHL5 were obtained from Addgene, U.S.A. UCHL3, UCHL5, and the catalytic domain of BAP1N (1–240) were subcloned into pGEX-6P-1 using standard cloning procedure. The pGEX-6P-1 UCHL1 plasmid was a gift from Dr. Chittaranjan Das, Purdue University, Department of Chemistry, U.S.A. Mutations in the catalytic domain of BAP1 were generated by site-directed mutagenesis using Quick-change Site Directed Mutagenesis Kit (Stratagene, Santa Clara, CA, U.S.A.). Catalytic domain BAP1N and all cancer-associated mutants were expressed in *Escherichia coli* Rosetta 2 cells (Novagen, Gibbs Town, NJ, U.S.A.) and proteins were purified according to the standard GST purification protocol. Briefly, Rosetta 2 cells containing pGEX-6P-1-BAP1N plasmid DNA were grown to 0.6 OD and then induced with 1 mM/l of IPTG at 18°C for 16 h. Cells were harvested and the pellet was suspended in 1× PBS with 400 mM KCl buffer containing 10 μg/ml lysozyme. Homogenized solution was loaded onto a GST column (GE Healthcare) that was previously equilibrated with 1× PBS with 400 mM KCl. Protein was eluted in 50 mM Tris/HCl, pH 8.0, 500 mM NaCl, and 20 mM GSH. GST tag was removed by Pre-Scission protease (GE Healthcare). Eluted protein was dialyzed extensively with 1× PBS with 400 mM KCl. Protein was concentrated and loaded onto HiLoad 16/600 Superdex 75 column (GE Healthcare) for gel filtration in 50 mM Tris/HCl, pH 7.4, 150 mM NaCl, and 5 mM DTT buffer. Eluted fractions were concentrated and purity was verified by SDS/PAGE.

### Determinations of the *K*_m_, *k*_cat_, and *k*_cat_/*K*_m_ values of Ub-AMC with BAP1

Multiple concentrations of Ub-aminomethylcoumarin (AMC) (100 nM–7.5 μM) (Boston Biochem Inc., Cambridge, MA, U.S.A.) were mixed in reaction buffer (50 mM Tris/HCl, 0.5 mM EDTA, 5 mM DTT, and 0.1% BSA) and constant concentration of BAP1N (250 pM final concentration) was added to initiate the enzymatic reaction (100 μl final volume). Rate of AMC released was monitored at 25°C with a SpectramaxM5 plate reader (Molecular Devices, U.S.A.) with excitation at 355 nm and emission at 455 nm. Initial velocity was calculated from the initial slope of each progress curve and *k*_cat_ and *K*_m_ values were determined from double-reciprocal plots assuming Michaelis–Menten model. The *K*_m_ value reported is the average of at least three independent measurements.

### Homology modeling

The homology-modeled BAP1N structure was generated in SWISS-MODEL [37] using UCHL5 crystal structure as a template. Further, the model was validated by online servers like PROCHECK [38,39] and Verify3D [40]. The molecular structure was visualized using PyMOL (PyMOL Molecular Graphics System, Schrödinger, LLC).

### Enzymatic activity assay

Stock solution of BAP1N and catalytic domain mutants E7Q, S10A, E31Q, Y33A, E148Q, I214A, T218A, R227A, F228H, and L230A were diluted with reaction buffer containing 50 mM Tris/HCl, pH 7.6, 5 mM DTT, 0.5 mM EDTA, 0.1% of BSA in the individual wells of 96-well plate to a final concentration of 250 pM. Ub-AMC (Boston Biochem Inc., Cambridge, MA, U.S.A.) was added to each well at a final concentration of 600 nM. Final reaction volume in each well was 100 μl. The rate of Ub-AMC hydrolysis was monitored at 25°C for 90 min using SpectramaxM5 plate reader (Molecular Devices, U.S.A.). The excitation and emission wavelength used in this measurement were 355 and 455 nm, respectively. The amount of AMC released due to hydrolysis by BAP1 was quantitated using 7-amido-4-methylcoumarin as a standard (Sigma–Aldrich). Effect of salt concentration on catalysis was monitored by using NaCl salt concentrations ranging from 0 to 1.5 M in the reaction buffer along with Ub-AMC in a final concentration of 450 nM.

### Surface plasmon resonance

Measurements of interactions using surface plasmon resonance (SPR) were carried out on a Biacore T200 (GE Healthcare). Ubiquitin in 10 mM sodium acetate with pH 4.5 was immobilized on to a dextran coated CM5 sensor chip (GE Healthcare) using a standard program, aiming for a density of 1250 response units (RU). Sensograms were collected at 25°C in 50 mM Tris at pH 7.4, 150 mM NaCl, and 0.5 mM DTT (GE Healthcare) at a flow rate of 30 μl/min, contact time of 120 s, and dissociation time of 600 s. Experiments were run in triplicate in the range of 0–50 μM for each BAP1 mutants dissolved in the same buffer. The binding affinity (*K*_D_) was calculated by steady state affinity binding model using Biacore T200 evaluation software (v. 1.0).

### Isothermal titration calorimetry

Isothermal titration calorimetry (ITC) experiments were performed using a Nano-ITC (TA Instruments, Newcastle, DE, U.S.A.) at 25°C. Ubiquitin from bovine erythrocytes was purchased from Sigma–Aldrich. Amino acid sequences between human and bovine ubiquitin are identical. Samples of UCHL1 C90S, UCHL3 C95S, UCHL5 C88S, and BAP1N C91S, and ubiquitin were extensively dialyzed against 50 mM Tris/HCl, pH 7.6, and 50 mM NaCl buffer which was then degassed to remove dissolved air. Enzyme concentration of 10 μM was used for UCHL1 C90S and UCHL5 C95S and ubiquitin concentration used for both was 100 μM. For UCHL5 C88S and BAP1N C91S concentrations used were 250 and 120 μM, respectively, ubiquitin used in the present study was ten-fold higher concentration than the respective enzyme concentrations. Titrations consisted of 2.5 μl of 20 injections of ubiquitin into the sample cell containing proteins with 5-min time intervals to ensure that each peak was returned to baseline. The heat of dilution was subtracted from each data by performing ubiquitin buffer titration experiment. All data were analyzed by the program included with the system (TA Instruments). The data were fitted to one-site binding model (one molecule of ubiquitin binding to one molecule of UCHs). Estimates of *K*_d_, ΔS, and ΔH were obtained by fitting the experimental data to the model and the best-fit parameters were selected based on the lowest chi-squared values.

### CD spectroscopy

CD spectra for wild-type and different mutants were recorded in the wavelength region from 260 to 190 nm. Briefly, wild-type and mutant BAP1N proteins were diluted in 50 mM phosphate buffer with pH 7.4 to a final concentration of 7 μM, and the spectra were recorded using JASCO J815 CD spectrometer at 25°C in wavelength scan mode. Typically, data were obtained as an average of ten scans by using a quartz cuvette of 0.2-cm path. The protein CD spectra were reported as the mean residue ellipticity (deg·cm^−2^·dmol^−1^). For determination of thermal melting point, wild-type and mutant BAP1N proteins were dissolved in 50 mM Tris/HCl, pH 7.4 to a final concentration of 7 μM. *T*_m_ for each protein was estimated from the unfolded protein fraction at 222 nm for varying temperature from 25 to 75°C maintained by the Peltier-type temperature controller.

## Results

### Kinetics of Ub-AMC hydrolysis by BAP1

To determine the kinetics of BAP1N-catalyzed hydrolysis, we performed a deubiquitinating assay using Ub-AMC as a substrate. A Michaelis–Menten saturation curve was plotted to determine the kinetics of catalysis for wild-type BAP1N. The calculated initial velocities of each substrate concentration were plotted against substrate concentrations ranging from 100 nM to 7.5 μM. The enzymatic parameters were estimated from double reciprocal plot to provide nonlinear least-squares parameters. Estimated Michaelis binding constant (*K*_m_) for BAP1N was ~2.9 μM which was 3.6-fold higher than UCHL5N (1–240) [41] (Table 1). BAP1N showed a higher affinity toward ubiquitin as compared with UCHL5N. However, it exhibited 99-fold and 149-fold less affinity toward ubiquitin than UCHL1 and UCHL3, respectively [42]. The turnover hydrolysis activity (*k*_cat_) for BAP1N was calculated to be 2.1 s^−1^, which was ~4-fold and 2.8-fold lower than hydrolysis rate of UCHL5N and UCHL3, respectively. BAP1N exhibited ~72-fold higher turnover rate than UCHL1. The *k*_cat_/*K*_m_ value defines the catalytic efficiency of an enzyme and the estimated efficiency of the BAP1N enzyme was 7.10 × 10^−5^ M^−1^s^−1^ that is comparable with UCHL5 and UCHL1. Interestingly, BAP1N exhibited 390-fold lower enzymatic efficiency than UCHL3, which suggests that BAP1 is not an efficient enzyme like UCHL3 (Table 1).

**Table 1:** Kinetic parameters of ubiquitin interaction with UCHs

Enzyme	*K*_m_ (µM)	*k*_cat_ (s^−1^)	*k*_cat_/*K*_m_ × 10^5^ (M^−1^ s^−1^)
UCHL1	0.03	0.03	8.87
UCHL3	0.02	5.9	2773
UCHL5 Cat (1–240)	10.7	8.7	8.10
BAP1N (1–240)	2.9 ± 0.6	2.1 ± 0.6	7.1 ± 0.5

Values for UCHL1, UCHL3 and UCHL5N are taken from literature [41,42].

### Homology modeling of the BAP1N structure

**Figure 1:**
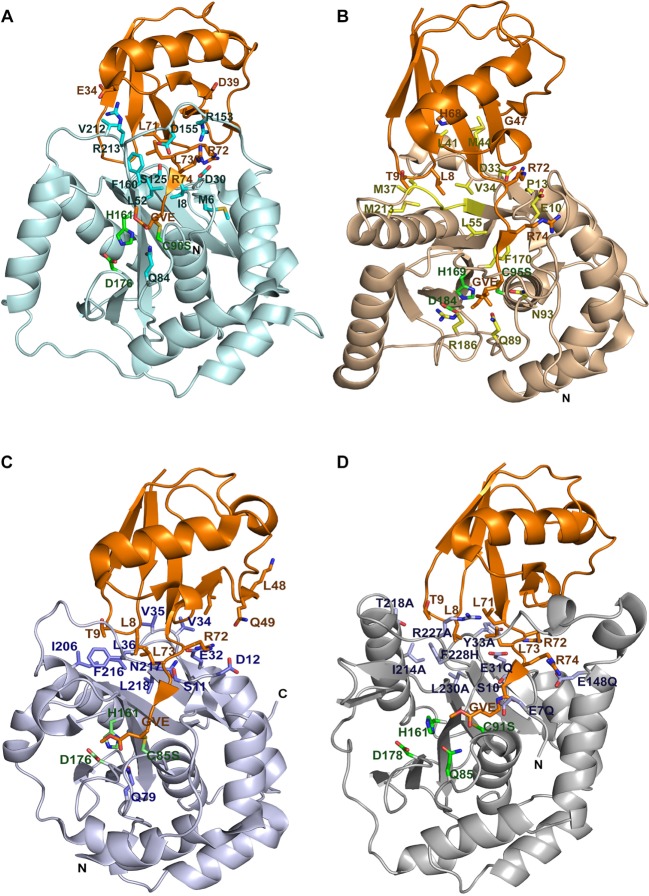
Structure of UCHs bound to the suicide substrate UbVMe Ribbon representation of the structure of UCHs–UbVMe complex (**A**) UCHL1 (3IFW) shown in pale cyan and residues interacting with ubiquitin are highlighted in cyan. (**B**) UCHL3 (1XD3) shown in wheat with residues interacting with ubiquitin highlighted in yellow (**C**) TsUCHL5 (4I6N) represented in light blue with residues interacting with ubiquitin highlighted in blue. (**D**) Structural mapping of predicted ubiquitin-interacting residues of BAP1 onto the UCHL5-based modeled structure of BAP1N in complex with UbVMe. BAP1N is colored gray, with predicted ubiquitin-interacting residues highlighted in black. In all structures UbVMe is shown in orange, catalytic residues are highlighted in green. Covalent bond formed between VMe and catalytic cysteine is shown in yellow. The panel is made using PyMOL (www.pymol.org).

Till date, there is no structural information available for BAP1 full length or domain wise. Thus the 3D model structure of BAP1N (5–240) was generated based on the coordinates of UCHL5N structure with the help of bioinformatics tool SWISS-MODEL [37]. Amongst the first three models generated, the one with the lowest energy was selected and docked with ubiquitin structure using PyMOL. The model was evaluated for protein geometry by PROCHECK (Ramachandran plot) and Verify3D [38–40]. The backbone dihedral distribution of all the amino acids calculated by Ramachandran plot highlighted that there were 98.6% of residues in favored regions. Model structure quality was checked by Verify3D [40]. Sequence alignment and structure comparison indicated that BAP1N shared a structural similarity with other UCH domains where the distance between catalytic triad residues was consistent with a canonical arrangement of the triad (Figure 1). The active site crossover loop of BAP1N (147–167, 21 amino acids) was longer than UCH-L5N (17 amino acids), UCH-L1 (11 amino acids), or UCH-L3 (14 amino acids) allowing substrate accessibility to its catalytic cleft and hydrolyze a large polyubiquitin chain. High degree of amino acid sequence diversity in the crossover loop of UCH members determined substrate processivity [36] (Supplementary Figure S1). It is interesting to note that the crossover loop sequence of individual UCHs is conserved amongst species, which indicates an evolutionary importance of crossover loop of a specific enzyme. Structural comparison of all UCHs confirms that an unproductive active site in their apo form is a common feature of UCH enzymes. UCHs undergo substrate-induced conformational changes upon ubiquitin binding to transform the active site from unproductive to productive form [43,44]. This might be true for BAP1 as well.

### Identification of specific interactions of ubiquitin with UCH domain of BAP1N

Currently, UCHL1 and UCHL3 crystal structures complexed with ubiquitin derivative of glycyl vinyl methyl ester (GlyVMe) have been reported [43,44] (Figure 1A,B). Crystal structures of human UCHL5N and full length have also been solved [41,45,46]. However, a ubiquitin-bound UCHL5 full-length structure is yet to be determined. Recently, *Ts*UCH37, a lower organism homolog of human UCHL5 is characterized from an infectious *Trichinella spiralis*, which shares 45% of its identity to that of human UCHL5 [47,48] (Figure 1C). We compared the interactions of ubiquitin with UCHL1, UCHL3, and *Ts*UCH37 and tried to reciprocate on to UCHL5-based homology model of BAP1N (Figure 1). The ubiquitin-bound homology model structure of BAP1N suggested that ubiquitin interacted at two sites on the enzyme. The C-terminus hexapeptide segment of ubiquitin made a contact with the active site cleft residues through numerous intermolecular interactions. The solvent-exposed hydrophobic crevice referred to as the distal site was occupied by two residues turn segment (Leu^8^–Thr^9^) of N-terminal β-hairpin of ubiquitin (Figure 1D). The solvent accessible surface area covered by ubiquitin in all UCHs was almost comparable, indicating a similar pattern of ubiquitin binding.

On the basis of ubiquitin-bound UCHs structures, residues involved in interactions were mapped onto the BAP1 sequence, by sequence alignment. Approximately ten residues E7, S10, E31, Y33, E148, I214, T218, R117, R228, and L230 were predicted to play an important role in BAP1N–ubiquitin interaction (Figure 1D and Supplementary Figure S1). Out of these ten proposed residues S10, E31, Y33, I214, F228, and L230, positions were chosen based on *Ts*UCHL5–ubiquitin interactions. T218, R227, F228, and L230 residues of BAP1N were selected based on ubiquitin interaction with the corresponding residues of UCHL1 and UCHL3. Two residues, E7 and E148 were selected on the basis of BAP1N model and ubiquitin interactions. S10, E31, I214, and L230 residues were conserved in BAP1N and UCHL5, while T218 was conserved between BAP1 and UCHL1, and Y33 was conserved in BAP1 and UCHL3 sequence. Residues R227 and F228 were conserved amongst all the UCHs and might have a critical role in ubiquitin interaction whereas E7 and E148 residues were unique to BAP1 sequence (Supplementary Figure S1).

### Probing the role of predicted residues in enzymatic activity

**Figure 2:**
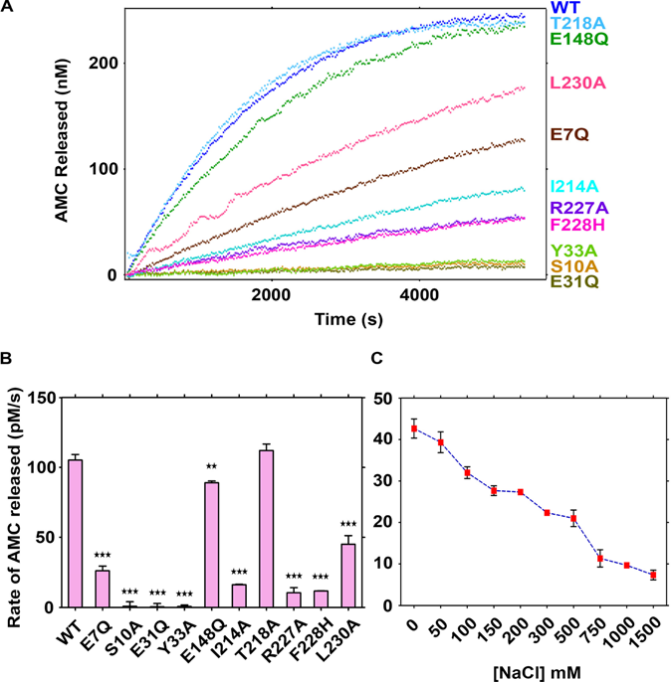
Hydrolysis of Ub-AMC catalyzed by BAP1N mutants Comparison of the enzymatic activity of wild-type BAP1N and the BAP1N variants E7Q, S10A, E31Q, Y33A, E148Q, I214A, T218A, R227A, F228H, and L230A. (**A**) Real-time reaction progress curves showing AMC released compared with time for the cleavage of Ub-AMC by wild-type BAP1N (blue) and BAP1N mutants are shown in different colors. The substrate and enzyme concentrations used for all reactions were 600 nM and 250 pM, respectively. (**B**) The rate of AMC hydrolysis calculated from initial slop of progress curve (*P*-value<0.05). (**C**) Dependence of enzyme velocity on the concentration of NaCl was determined for wild-type BAP1N. At each concentration of NaCl, values of initial velocities were calculated in triplicate. Final enzyme and substrate concentration in 100-μl reaction volume were 250 pM and 450 nM, respectively.

Interestingly, S10, E31, Y33, R227, and L230 residues of BAP1 were found to be mutated in many cancers as specified in the COSMIC database. We have recently reported that cancer-associated BAP1N mutants are enzymatically inactive and are prone to structural destabilization and aggregation [14]. Considering our earlier observation, one can argue that these five residues may be very crucial for ubiquitin interactions or they are important in structural integrity and both these factors contribute to the inactivation of deubiquitinating activity. To rule out the possibility of structural instability of BAP1N due to mutation, we used charge neutralizing amino acid substitution to study the importance of predicted residues in BAP1N–ubiquitin interaction. After mutagenesis and purification of wild-type BAP1N and mutant proteins (E7Q, S10A, E31Q, Y33A, E148Q, I214A, T218A, R227A, F228H, and L230A), steady-state kinetic experiments were performed using Ub-AMC as a substrate in 50 mM Tris/HCl, pH 7.6, 5 mM DTT, 0.5 mM EDTA, and 0.1% of BSA reaction buffer. Most of the mutants showed impaired catalytic activity in comparison with wild-type BAP1N (Figure 2A). Mutants S10A, E31Q, and Y33A were unable to hydrolyze Ub-AMC substrate whereas I214A, R227A, and F228H showed almost 6.5-, 10- and 9-fold lower AMC release rate than wild-type BAP1N, respectively. E7Q and L230A showed moderate activity with 4- and 2.3-fold decrease in Ub-AMC hydrolysis, respectively. On the other hand, T218A and E148Q mutants did not show any change in the rate of AMC release compared with wild-type BAP1N (Figure 2B, Supplementary Table S1). All the Ub-AMC hydrolysis experiments were carried out in the absence of NaCl. Effects of salt concentration on the rate of hydrolysis of Ub-AMC by wild-type BAP1N and mutants were studied. The catalytic activity of wild-type BAP1N and mutants reduced significantly in 150 mM NaCl concentration (Supplementary Figure S2) and activity of wild-type BAP1N gradually decreased with increasing concentration of NaCl (Figure 2C). Our results corroborate with the studies where it has been shown that *K*_m_ of UCHL1 and USP7 for Ub-AMC substrate significantly increases in higher salt concentration [49,50].

**Figure 3:**
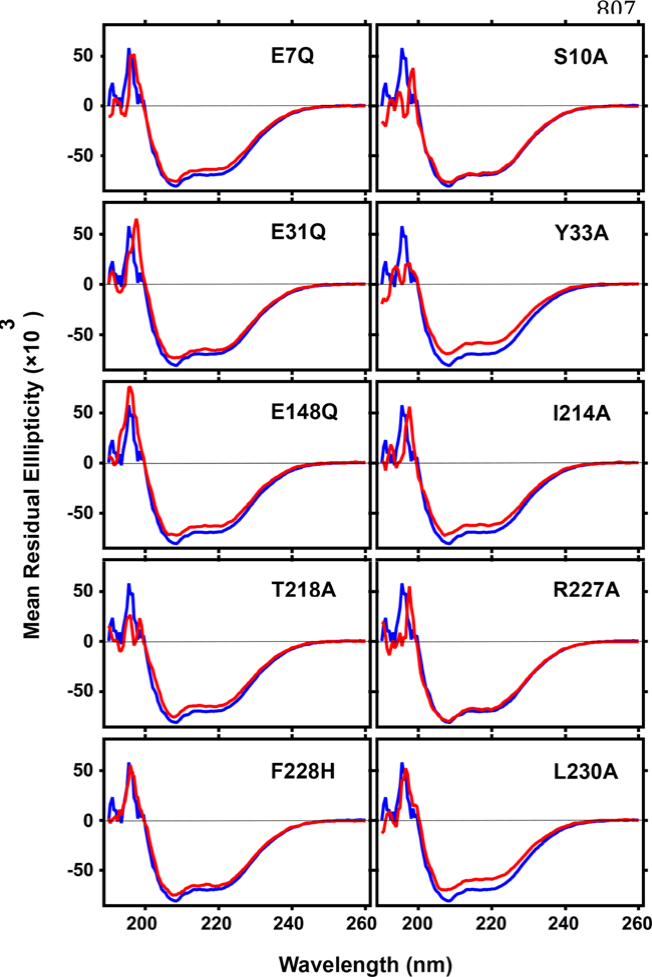
Structural studies of BAP1N carrying the mutations CD spectra at 25°C of wild-type BAP1N and catalytic domain mutants showed characteristic secondary structure between 190 and 260 nm. The blue curve represents wild-type BAP1N and ubiquitin interaction specific mutants are shown in red.

**Figure 4:**
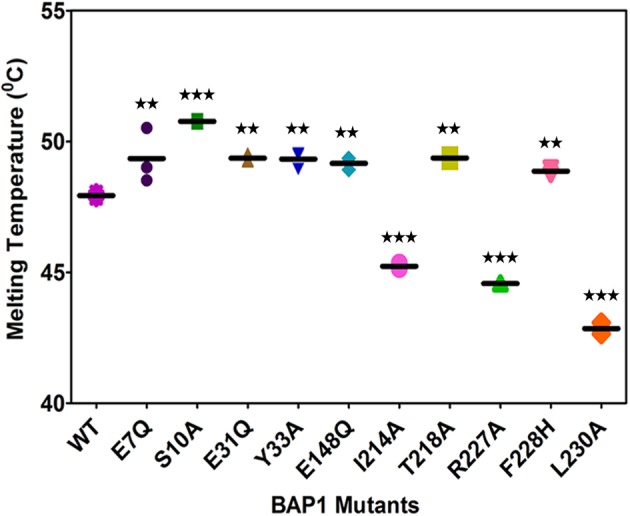
Change in ellipticity at 222 nm as a function of temperature CD parameters for thermally induced unfolding of wild-type BAP1N and mutants were measured. *T*_m_ is the midpoint of the thermal transition unfolding over the range of 25–75°C, which was calculated from sigmoidal fitting in IGOR software. The values are mean ± S.D. of at least three experiments (*P*-value <0.05).

Inhibition of enzymatic activity of the mutants prompted us to further investigate the effect of mutation on protein structure. Protein secondary structure was examined by CD spectroscopy. No significant differences were found in the ellipticity of mutants compared with wild-type BAP1N except Y33A and T218A. Secondary structure analysis suggested minor mutational effects on the structure of most of the mutant proteins (Figure 3, Supplementary Table S2). The thermally induced unfolding was monitored to validate the involvement of selected amino acids in the intramolecular interaction in the global structural stability of mutants (Supplementary Figure S3). *T*_m_ for most of the mutants except R227A and L230A was within ±3°C of wild-type BAP1N suggesting their role only in intermolecular interactions. *T*_m_ of R227A and L230A mutants was 3.3 and 5.1°C lower than BAP1N, wild-type, respectively indicating that these residues play an important role in maintaining the structural scaffold (Figure 4, Supplementary Table S3).

### Ubiquitin affinity measurements of predicted residues

**Figure 5:**
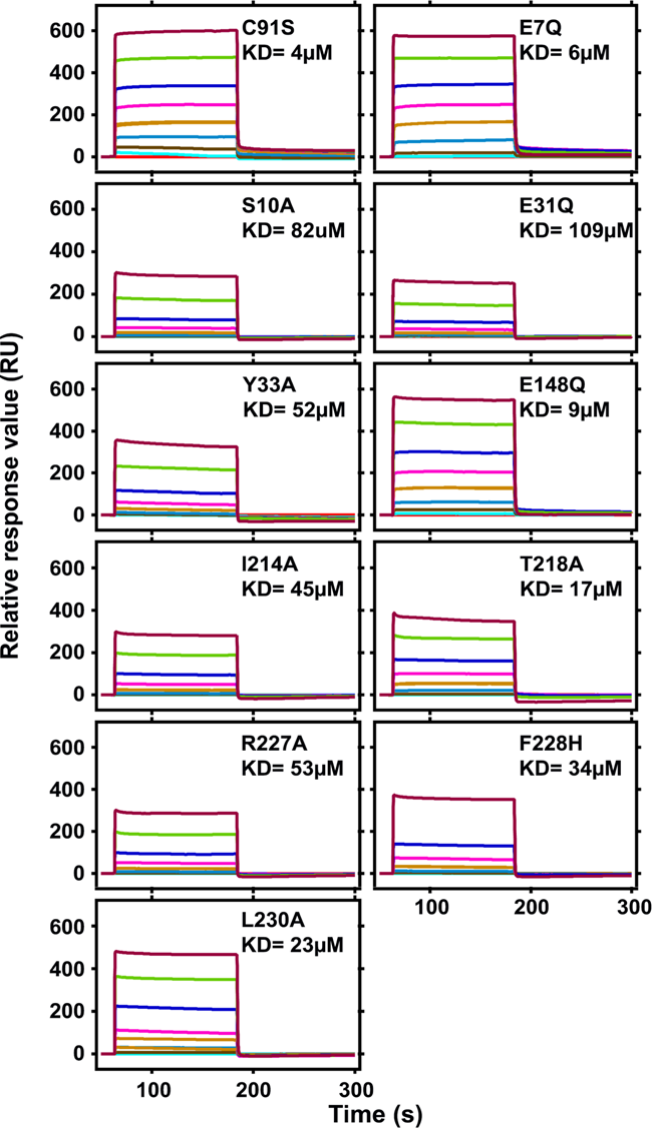
Sensograms for the binding of BAP1N mutants to ubiquitin BAP1N C91S and other double mutants of concentration ranging from 0 to 50 μM in 50 mM Tris, pH 7.4 and 150 mM NaCl were perfused over ubiquitin immobilized covalently on a hydrophilic carboxymethyl-dextran matrix (Sensor Chip CM5). Representative sensograms show binding profiles of all the proteins.

Catalytic inactive BAP1N double mutants were made by site-directed mutagenesis to check if mutations contributed significantly to ubiquitin binding. Catalytic cysteine at position 91 was replaced by serine (C91S) in all the mutants to make it catalytically inactive. The secondary structure analysis of all double mutants was checked by CD spectra analysis. The double mutation did not perturb the overall structure of BAP1N mutants (Supplementary Figure S4) making it an ideal system for binding analysis. Binding experiments were executed by SPR to measure the affinity of ubiquitin for BAP1N double mutants. The ubiquitin was immobilized on a dextran-coated CM5 chip and probed for BAP1N double mutants binding ability at pH 7.4. Time-dependent binding and dissociation of BAP1N mutants to ubiquitin at varied protein concentrations were shown in Figure 5. The linear increase and quick saturation of the resonance signal indicated that the association rate constant was very high and that dissociation was also very fast suggesting a strong affinity but a transient interaction between two proteins. The binding constant (*K*_D_) of BAP1N C91S for ubiquitin was 4 ± 1.6 µM (Supplementary Table S4). The ubiquitin affinity for E7Q and E148Q did not change significantly while catalytically inactive mutants S10A, E31Q, and Y33A showed 21-, 27-, and 13-fold decrease in ubiquitin binding. Rest of the mutants I214, R227A, F228H, and L230A showed 11-, 13-, 9-, and 6-fold lower binding affinity toward ubiquitin, respectively. Interestingly, T218A, which was catalytically as efficient as wild-type BAP1N, showed ~four-fold decreased affinity toward ubiquitin (Supplementary Table S4).

### Thermodynamics of BAP1N–ubiquitin interaction and comparison with other UCHs

**Figure 6:**
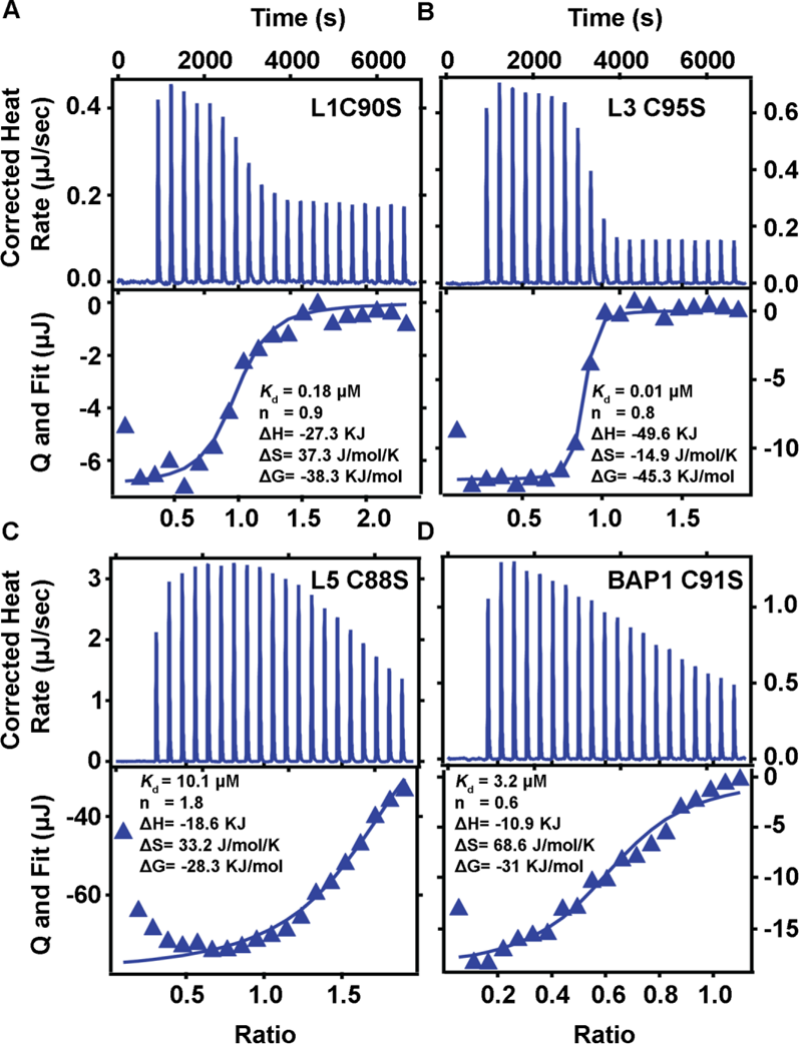
Binding isotherm of the titration of catalytic mutants of UCHL1, UCHL3, UCHL5, and BAP1N with ubiquitin Data acquisition and quantitative analysis of the ubiquitin binding of UCHs were performed on TA-ITC instrument. ITC profile for the binding of ubiquitin to (**A**) UCHL1 C90S (10 μM) with ubiquitin (100 μM), (**B**) UCHL3 C95S (10 μM) with ubiquitin (100 μM), (**C**) UCHL5 C88S (250 μM) with ubiquitin (2.5 mM) and (**D**) BAP1N C91S (120 μM) with ubiquitin (1.2 mM) was obtained at 25°C. The top panel for each figure shows the raw data, and the lower panel shows the integrated heat data as enthalpy as a function of molar ratio of ligand to protein. The solid line in the bottom panel represents the best curve fit to the data by using one-site binding model.

ITC was performed to examine the thermodynamics of ubiquitin interaction with BAP1N. The energetics of ubiquitin–UCHs interactions were also estimated. Catalytic inactive mutant of BAP1 (C91S), UCHL5 (C88S), UCHL3 (C95S), and UCHL1 (C90S) were titrated with ubiquitin in ITC at 25°C (Figure 6). The thermodynamics of ubiquitin binding to all four UCH family members were shown in Table 2. The binding was evident with an exothermic reaction. All the binding curves were fitted with a one-site binding model to get binding constant, enthalpy, entropy, and free energy of interaction. Binding constant (*K*_d_) for BAP1N (C91S) and ubiquitin interaction was 3.2 µM at pH 7.4, which was consistent with *K*_m_ value obtained from the enzymatic kinetics analysis and SPR binding experiment (Table 1 and Supplementary Table S4). The thermodynamic parameters *K*_d_ ~3.2 µM, ΔH ~ –10.9 kJ/mol, ΔS ~68.6 J/mol·K, and ΔG ~ –31 kJ M^−1^ for BAP1N C91S and ubiquitin were generated from the binding analysis. An earlier attempt to study the thermodynamics of ubiquitin binding to full-length UCHL5 in its apo form was not effective because of the low concentration of enzyme and substrate. However, it is reported that the binding of RPN13^DEU^ to ULD of UCHL5 increases the affinity of UCHL5 to ubiquitin giving *K*_d_ value of 4.5 µM, ΔH ∼ –18.3 kcal/mol, TΔS ∼ –10.96 kcal/mol [29]. Indeed, our binding studies by SPR showed a high *K*_D_ value of 20.1 µM for free full-length UCHL5 C88S with ubiquitin (Supplementary Figure S5). Accordingly, higher concentrations of UCHL5 C88S (250 µM) and ubiquitin (2.5 mM) were used in the present ITC study. The binding parameters for UCHL5 and ubiquitin interaction were *K*_d_ ~10.1 µM, ΔH ~ –18.6 kJ/mol, ΔS ~33.2 J/mol·K, and ΔG ~ –28.3 kJ M^−1^. Thermodynamic parameters of UCHL3–ubiquitin interactions were found to be *K*_d_ value of ~0.01 µM, ΔH ~ –49.6 kJ/mol, ΔS ~ –14.9 J/mol·K, and ΔG ~ –45.3 kJ M^−1^ (Table 2) which was consistent with the *K*_m_ value reported earlier.

**Table 2:** Binding and thermodynamic parameters for UCHL1 C90S, UCHL3 C95S, UCHL1 C88S, and BAP1 C91S in 50 mM Tris, pH 7.4 and 50 mM NaCl

Parameters	Proteins			
	L1 C90S	L3 C95S	L5 C88S	BAP1 C91S
ΔH (kJ/mol)	–27.3 ± 0.5	–49.6 ± 1.6	–18.6 ± 1.3	–10.9 ± 1.1
*n*	0.9 ± 0.1	0.8 ± 0.02	1.2 ± 0.1	0.6 ± 0.4
*K*_d_ (μM)	0.2 ± 0.1	0.01 ± 0.001	10.1 ± 3.6	3.2 ± 1.0
ΔS (J/mol·K)	37.3 ± 4.4	–14.9 ± 5.6	33.2 ± 0.5	68.6 ± 2.7
ΔG (kJ M^−1^)	–38.3 ± 0.7	–45.3 ± 0.3	–28.3 ± 1.2	–31 ± 0.6

*K*_d_, dissociation constant; *n*, binding stoichiometry; ΔH, change in enthalpy; ΔS, change in entropy.

## Discussion

In the present study, we have investigated the enzymatic properties of BAP1N and compared it with other UCH members. We have used the catalytic domain of BAP1 for our study as full-length BAP1 protein expression is very less compared with the catalytic domain and our earlier report has demonstrated that the truncation of a middle and C-terminal domain of BAP1 does not lower the catalytic activity rather BAP1N shows higher activity. It is hypothesized that the C-terminal ULD domain of BAP1 blocks catalytic cleft of BAP1 making it a less efficient enzyme [14]. According to our analysis, UCHL3 shows the highest enzymatic efficiency with low *K*_m_ and high *k*_cat_ values amongst all the UCH members. However, UCHL1 is considered to be a poor enzyme despite its comparable *K*_m_ with UCHL3 due to a misaligned active site and compromised *k*_cat_. The catalytic efficiency of UCHL5N is comparable with the UCHL1 mainly because of its high *k*_cat_ contribution. BAP1N shows a very much similar catalytic efficiency (*k*_cat_/*K*_m_) to UCHL5. Similar catalytic efficiencies of both enzymes correlate well with a support of common ancestry for BAP1 and UCHL5 regulators in PR–DUB and INO80 chromatin remodeling complexes.

An important finding of our work is the identification of residues of BAP1N playing a crucial role in ubiquitin interaction. Some of the mutants have shown a varying degree of catalytic activity impairment and a few mutants have shown complete loss of activity. The catalytic activity of all mutants has suggested that either these residues are crucial in ubiquitin interaction or they are essential for structural stability. We have reported previously that cancer-associated BAP1N mutants show reduced structure stability and increased aggregation propensity [14]. The complete loss of catalytic activity of S10A, E31Q, and Y33A indicate that these residues are essential for structural stability.

**Figure 7:**
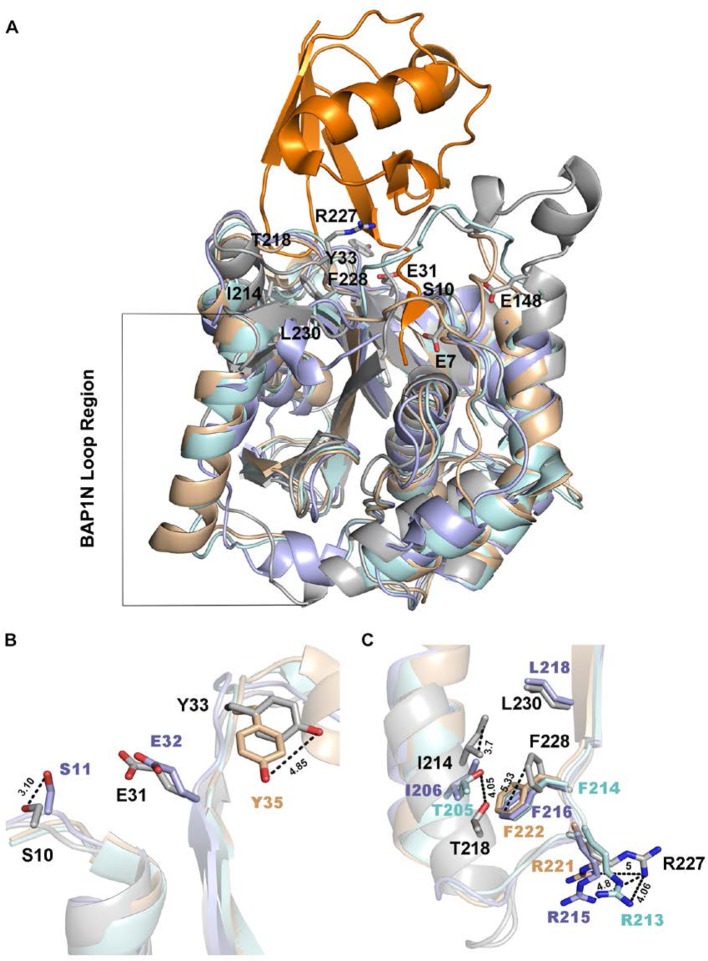
Superposition of UCHL1, UCHL3, *Ts*UCH37 X-ray structures with BAP1N model structure (**A**) Overall view of UCHs superimposed structures with ubiquitin in orange. UCHL5-based BAP1N model structure is shown in gray, UCHL1 is shown in pale cyan, UCHL3 in wheat, and TsUCH37 in light blue. Predicted BAP1 residues interacting with ubiquitin are labeled in black. (**B**) A close-up view of S10, E31, and Y33 of BAP1 aligned with their corresponding residues in other UCHs. S10 and Y33 show deviation of 3 and 4.9 Å from S11 and Y35 of *Ts*UCH37 and UCHL3 respectively. E31 of BAP1 does not show any deviation from E32 of *Ts*UCH37. (**C**) The shifts in the position of BAP1 I214 and T218 from I206 of *Ts*UCH37 and T205 of UCHL1 are around 3.7 and 4.1 A° respectively. Change in the orientation of F228 of BAP1 from F216 of *Ts*UCH37, F214 of UCHL1 and F222 of UCHL3 was ~5 Å. Deviations in the orientation of R227 from R213, R215 and R221 are ~4, 4.8, and 5 Å, respectively. L230 of BAP1 does not show any deviation from its corresponding residue in *Ts*UCH37.

The superposition of BAP1N homology model with the crystal structure of UCHL1, UCHL3, and *Ts*UCH37 shows similar bilobal architecture where a major change is observed from E51 to D75 with the presence of loop instead of a helix (Figure 7A). Amongst the residues chosen for the study, a major difference can be seen for the conformation of S10, Y33, I214, T218, R227, and F228 in comparison with corresponding residues on UCHL1, UCHL3, and *Ts*UCH37. A notable difference in side chain orientation was found for S10, Y33, F228, and R227 residues whereas I214 and T218 displayed spatial difference. No alteration was observed in confirmation of E31 and L230 residues compared with E32 and L218 of *Ts*UCH37, respectively (Figure 7B,C).

The thermodynamic analysis reveals that BAP1–ubiquitin interaction is entropically driven. Entropy-driven interactions are majorly associated with hydrophobic interactions and a large amount of static water displacement upon ubiquitin binding. Large structural rearrangement upon ubiquitin binding may contribute to increased entropy. An earlier report has suggested that ASXL1 binding to BAP1 decreases the entropy significantly [32] and stabilizes the BAP1–ASXL1 complex. In contrast with BAP1, the thermodynamics of ubiquitin binding to UCHL1 and UCHL3 is dominated by enthalpy factors and hydrogen bonding and ionic interactions that stabilize the complex. Indeed, both UCHL1–UbVMe and UCHL3–UbVMe complexes are associated with large numbers of hydrogen bonding and ionic interactions as evident from crystallographic data.

Our comprehensive biochemical analysis of ubiquitin binding to UCHs provides a framework for future work on the possible role of divergence in ubiquitin interaction despite having conserved sequences in these UCHs. Clearly, the present study offers an opportunity to test this model and proposed sites for BAP1–ubiquitin interaction analysis. The molecular insight into BAP1–ubiquitin complex will assist researchers to design a potent inhibitor of this enzyme.

## Supporting information

**Supplementary Figure S1:** **Multiple sequence alignment of BAP1 with human UCHL5, UCHL1, UCHL3 and TsUCH37.** Structure based sequence alignment of UCH domain of BAP1 with other known human UCHs and *Trichinella spiralis* ubiquitin hydrolase *Ts*UCH37. Catalytic residues of BAP1 (blue colored asterisks), conserved among the UCHs and *Ts*UCH37, are highlighted in blue. Residues highlighted in yellow are important for ubiquitin binding, curated from the ubiquitin bound crystal structure data of human UCHL1, UCHL3, UCHL5 and *Ts*UCHL5. The proposed BAP1N residues interacting with ubiquitin are shown with red colored asterisks and similar residues in other UCHs highlighted in red. Active site crossover loop residues are highlighted in green.

**Supplementary Figure S2:** **Effect of salt on kinetics of Ub-AMC hydrolysis.** Rate of Ub-AMC hydrolysis by wild-type BAP1N and catalytic domain mutants E7Q, S10A, E31Q, Y33A, E148Q, I214A, T218A, R227A, F228H and L230A in without salt reaction buffer (50 mM Tris-HCl, pH 7.6, 5 mM DTT, 0.5 mM EDTA, 0.1% of BSA) shown in pink and with salt (50 mM Tris-HCl, pH 7.6, 150 mM NaCl, 5 mM DTT, 0.5 mM EDTA, 0.1% of BSA) is shown in red. The values are the mean ± standard deviation of at least two experiments (P value < 0.05).

**Supplementary Figure S3:** **Thermal stability of mutants by CD.** Complete temperature unfolding profile at 222 nm demonstrated distinct unfolding nature of wild-type BAP1N shown in red and mutants shown in blue. Melting temperatures were obtained from sigmoidal fits over the range of 25 °C to 75 °C controlled by Peltier control system using 0.2 cm path length cuvette. The protein concentration was 7 μM in 50 mM Tris, pH 7.4 and 150 mM NaCl. Solid line represents the sigmoidal curve fitting.

**Supplementary Figure S4:** **Secondary Structure studies of BAP1N carrying double mutations.** Circular dichroism spectra at 25 °C of BAP1N C91S and proposed ubiquitin interacting residues of BAP1N with C91S double mutation showed similar characteristic secondary-structure between 190–260 nm as that of BAP1N C91S. Black curves represents BAP1N C91S and ubiquitin interaction specific double mutants are shown in red.

**Supplementary Figure S5:** **Sensograms for the binding of UCHL5 C88S with ubiquitin.** UCHL5 C88S of concentration ranging from 0-50 μM in 50 mM Tris, pH 7.4 and 150 mM NaCl were perfused over ubiquitin immobilized covalently on a CM5 Sensor Chip. Representative sensograms show binding profile of UCHL5 C88S protein.

**Supplementary Table 1:** Rate of Ub-AMC Hydrolysis by wild-type BAP1N and its mutants.

**Supplementary Table 2:** Secondary structure analysis of BAP1N single mutants

**Supplementary Table 3:** Thermal melting point of wild-type BAP1N and its mutants.

**Supplementary Table 4:** SPR-derived binding constants for the interaction between different BAP1N double mutants and immobilized ubiquitin.

## Abbreviations

AMC: aminomethylcoumarin
ASXL: Additional sex comb-like
BAP1: BRCA1-associated protein 1
BAP1N: N-terminal catalytic domain of BAP1
BARD1: BRCA1 associated RING domain 1
BRCA1: Breast cancer 1
COSMIC: Catalogue of Somatic Mutations in Cancer
FOXK2: Forkhead box K2
ITC: isothermal titration calorimetry
MBD: Methyl CpG binding domain
OD: Optical density
OGT: O-linked β-N-acetylglucosamine transferase
PR-DUB: Polycomb repressive complex
SPR: surface plasmon resonance
UCH: Ubiquitin C-terminal hydrolase
UCHL1: ubiquitin C-terminal hydrolase L1
UCHL3: ubiquitin C-terminal hydrolase L3
UCHL5: ubiquitin C-terminal hydrolase L5
ULD: UCHL5-like domain
