# Supplementary material for: Ubiquitin recognition of BAP1: understanding its enzymatic function
Source: Biosci Rep. 2017 Oct 27;37(5):BSR20171099. doi: 10.1042/BSR20171099 (PMC5665613; doi:10.1042/BSR20171099)
Supplement: Supplementary file 1 [file BSR20171099_Supp1.pdf]

Supplementary Information

Ubiquitin Recognition of BAP1: Understanding its Enzymatic Function

Pranita Hanpude [1, 2], Sushmita Bhattacharya [1], Abhishek Kumar Singh [1] and Tushar Kanti Maiti [1*]

[1] Laboratory of Functional Proteomics, Regional Centre for Biotechnology, NCR Biotech Science Cluster, 3rd Milestone Gurgaon-Faridabad Expressway, Faridabad, Haryana 121001, INDIA.

[2] Manipal University, Karnataka, 576104, INDIA.

* Correspondence and requests for materials should be addressed to Tushar K. Maiti. (Email: tkmaiti@rcb.res.in)

Supplementary Figure legends:

Supplementary Figure S1. Multiple sequence alignment of BAP1 with human UCHL5, UCHL1, UCHL3 and *Ts*UCH37. Structure based sequence alignment of UCH domain of BAP1 with other known human UCHs and *Trichinella spiralis* ubiquitin hydrolase *Ts*UCH37. Catalytic residues of BAP1 (blue colored asterisks), conserved among the UCHs and *Ts*UCH37, are highlighted in blue. Residues highlighted in yellow are important for ubiquitin binding, curated from the ubiquitin bound crystal structure data of human UCHL1, UCHL3, UCHL5 and *Ts*UCHL5. The proposed BAP1N residues interacting with ubiquitin are shown with red colored asterisks and similar residues in other UCHs highlighted in red. Active site crossover loop residues are highlighted in green.

Supplementary Figure S2. Effect of salt on kinetics of Ub-AMC hydrolysis. Rate of Ub-AMC hydrolysis by wild-type BAP1N and catalytic domain mutants E7Q, S10A, E31Q, Y33A, E148Q, I214A, T218A, R227A, F228H and L230A in without salt reaction buffer (50 mM Tris-HCl, pH 7.6, 5 mM DTT, 0.5 mM EDTA, 0.1% of BSA) shown in pink and with salt (50 mM Tris-HCl, pH 7.6, 150 mM NaCl, 5 mM DTT, 0.5 mM EDTA, 0.1% of BSA) is shown in red. The values are the mean ± standard deviation of at least two experiments (P value < 0.05).

Supplementary Figure S3. Thermal stability of mutants by CD. Complete temperature unfolding profile at 222 nm demonstrated distinct unfolding nature of wild-type BAP1N shown in red and mutants shown in blue. Melting temperatures were obtained from sigmoidal fits over the range of 25 °C to 75 °C controlled by Peltier control system using 0.2 cm path length cuvette. The protein concentration was 7 μM in 50 mM Tris, pH 7.4 and 150 mM NaCl. Solid line represents the sigmoidal curve fitting.

Supplementary Figure S4. Secondary Structure studies of BAP1N carrying double mutations. Circular dichroism spectra at 25 °C of BAP1N C91S and proposed ubiquitin interacting residues of BAP1N with C91S double mutation showed similar characteristic secondary-structure between 190–260 nm as that of BAP1N C91S. Black curves represents BAP1N C91S and ubiquitin interaction specific double mutants are shown in red.

Supplementary Figure S5. Sensograms for the binding of UCHL5 C88S with ubiquitin. UCHL5 C88S of concentration ranging from 0-50 μM in 50 mM Tris,

pH 7.4 and 150 mM NaCl were perfused over ubiquitin immobilized covalently on a CM5 Sensor Chip. Representative sensograms show binding profile of UCHL5 C88S protein.

Supplementary Figure S1

```
              *  *                     * *
1    ---MNKGWLELESDPGLFTLLVEDFGVKGV-QVEEIYDLQSK----CQGPVYGFIFLFKW 52   Q92560     BAP1_HUMAN
1    MTGNAGEWCLMESDPGVFTELIKGFGCRGA-QVEEIWSLEPENFE-KLKPVHGLIFLFKW 58   Q9Y5K5     UCHL5_HUMAN
1    --MAEGNWCLIESDPGIFTEMIHGFGCTGL-QVEELVVLD-ESIE-HLKPIHGFIFLFRW 56   A0A0V0WDD0 UCHL5_Trichinella Sp.
1    -----MQLKPMEINPEMLNKVLSRLGVAGQWRFVDVLGLEEESLGSVPAPACALLLLFPL 55   P09936     UCHL1_HUMAN
1    --MEGQRWLPLEANPEVTNQFLKQLGLHPNWQFVDVYGMDPELLSMVPRPVCAVLLLFPI 58   P15374     UCHL3_HUMAN

                                     *     *
53   IEERRSRRKVSTLVDDTSVIDDDIVNNMFFAHQLIPNSCATHALLSVLLNCS-S--VDLG 109  Q92560     BAP1_HUMAN
59   QPGEEPA---------GSVVQDSRLDTIFFAKQVINNACATQAIVSVLLNCT-HQDVHLG 108  Q9Y5K5     UCHL5_HUMAN
57   LKKEMRK---------EVDDSPQTCTDVYFSQQVIQNACASQALINLLLNCD-HPDVDLG 106  A0A0V0WDD0 UCHL5_Trichinella Sp.
56   TAQHENFRKKQI--EEL--KGQEVSPKVYFMKQTIGNSCGTIGLIHAVANNQDKLGFEDG 111  P09936     UCHL1_HUMAN
59   TEKYEVFRTEEE--EKIKSQGQDVTSSVYFMKQTISNACGTIGLIHAIANNKDKMHFESG 116  P15374     UCHL3_HUMAN

                                           *                    *
110  PTLSRMKDFTKGFSPESKGYAIGNAPELAKAHNSHARPEPRHLPEKQNGLSAVRTMEAFH 169  Q92560     BAP1_HUMAN
109  ETLSEFKEFSQSFDAAMKGLALSNSDVIRQVHNSFARQQMFEFDTK----TSAKEEDAFH 164  Q9Y5K5     UCHL5_HUMAN
107  PTLKEFKDFTYDLDSASRGLCLTNSEKIRAVHNSFGRQQLFEIDDQ----QKLDEEDVFH 162  A0A0V0WDD0 UCHL5_Trichinella Sp.
112  SVLKQFLSETEKMSPEDRAKCFEKNEAIQAAHDAVAQEGQC----------RVDDKVNFH 161  P09936     UCHL1_HUMAN
117  STLKKFLEESVSMSPEERARYLENYDAIRVTHETSAHEGQTE-------APSIDEKVDLH 169  P15374     UCHL3_HUMAN

                   *                              *   *        **
170  FVSYVPITGRLFELDGLKVYPIDHGPWG-EDEEWTDKARRVIMERIGLATAGEPYHDIRF 228  Q92560     BAP1_HUMAN
165  FVSYVPVNGRLYELDGLREGPIDLGACN-Q-DDWISAVRPVIEKRIQKYSE----GEIRF 218  Q9Y5K5     UCHL5_HUMAN
163  FVTYVPVNDGVYELDGLRAAPLRLGTVASD-GDWTEVAIKAIKEKIKNYGE----SEVRF 216  A0A0V0WDD0 UCHL5_Trichinella Sp.
162  FILFNNVDGHLYELDGRMPFPVNHGASS-EDTLLKDAAK-VC----REFTE-REQGEVRF 214  P09936     UCHL1_HUMAN
170  FIALVHVDGHLYELDGRKPFPINHGETS-DETLLEDAIE-VC----KKFME-RDPDELRF 222  P15374     UCHL3_HUMAN

      *
229  NLMAVVPDRRIK------------------------------------------------ 240  Q92560     BAP1_HUMAN
219  NLMAIVSDRKMI------------------------------------------------ 230  Q9Y5K5     UCHL5_HUMAN
217  NLMAVISDQKLK------------------------------------------------ 228  A0A0V0WDD0 UCHL5_Trichinella Sp.
215  SAVALCKAA--------------------------------------------------- 223  P09936     UCHL1_HUMAN
223  NAIALSAA---------------------------------------------------- 230  P15374     UCHL3_HUMAN
```

Supplementary Figure S2

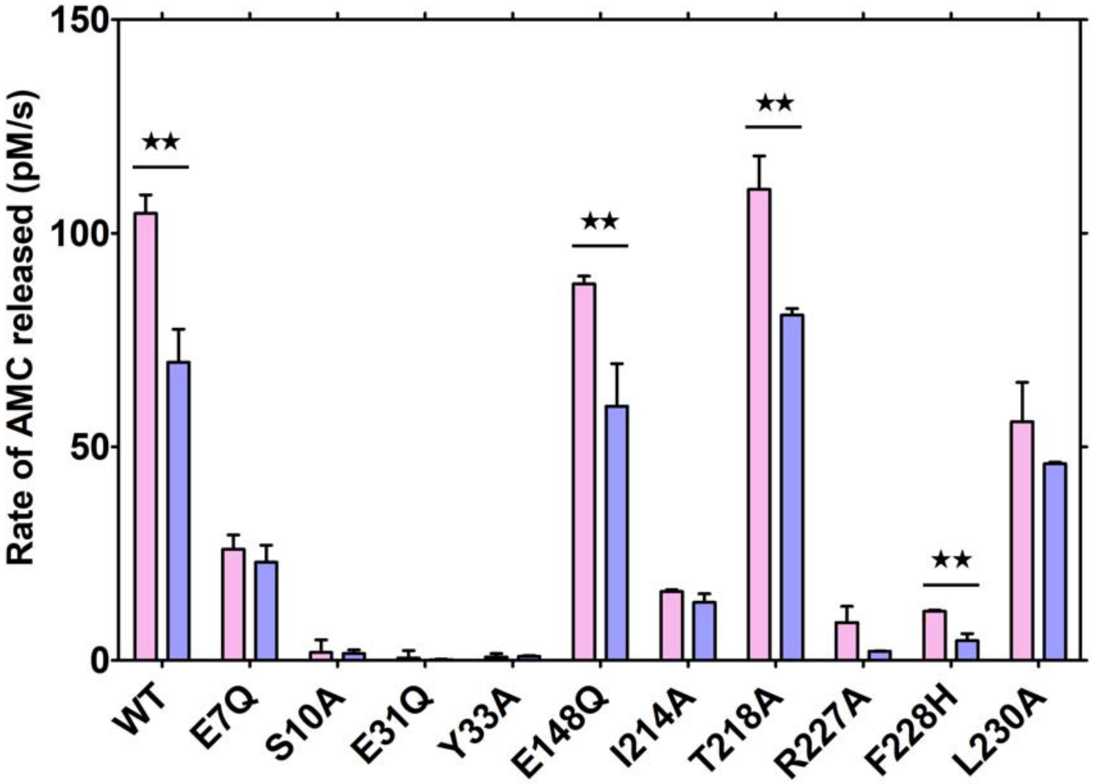

Supplementary Figure S3

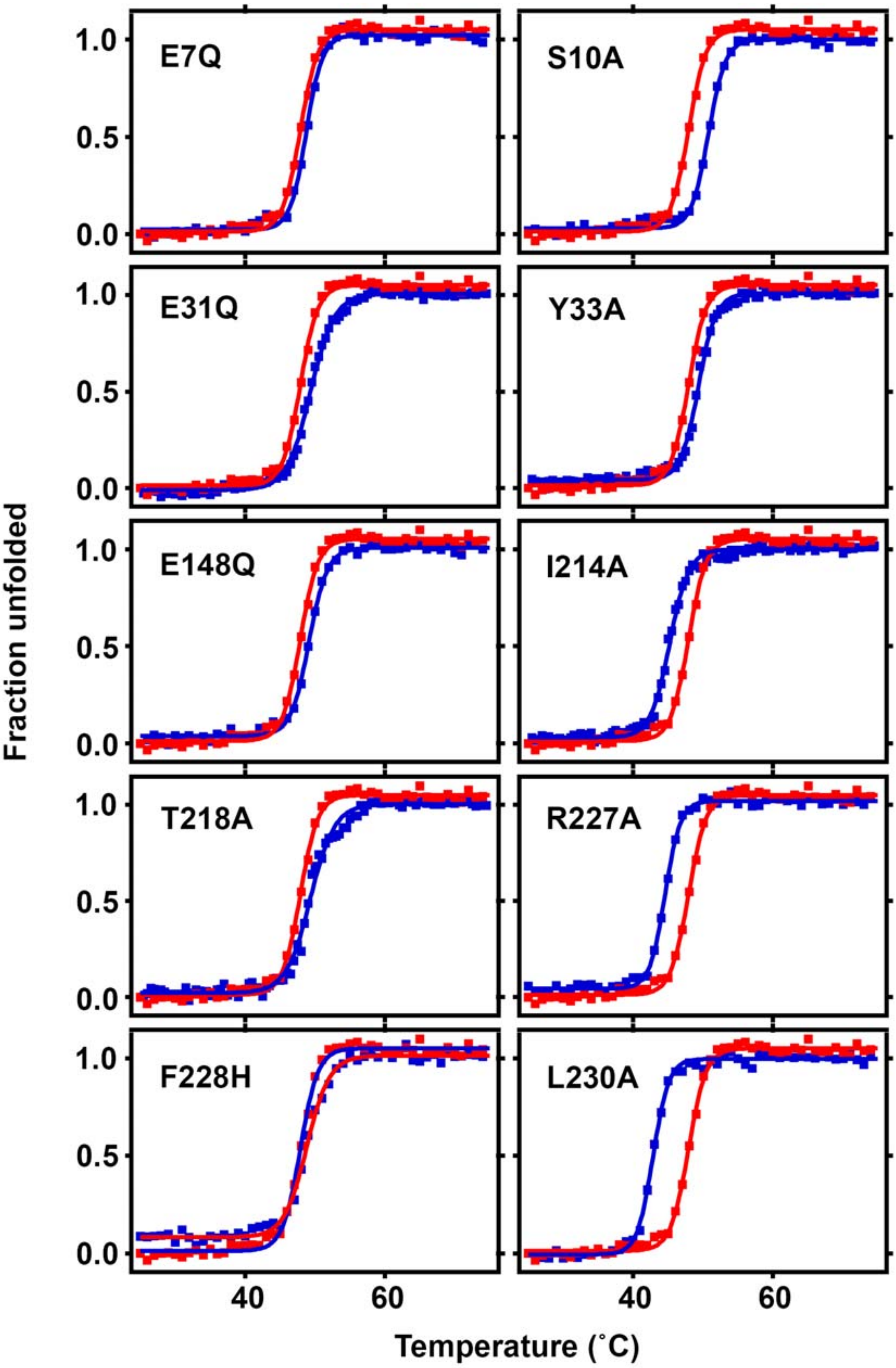

Supplementary Figure S4

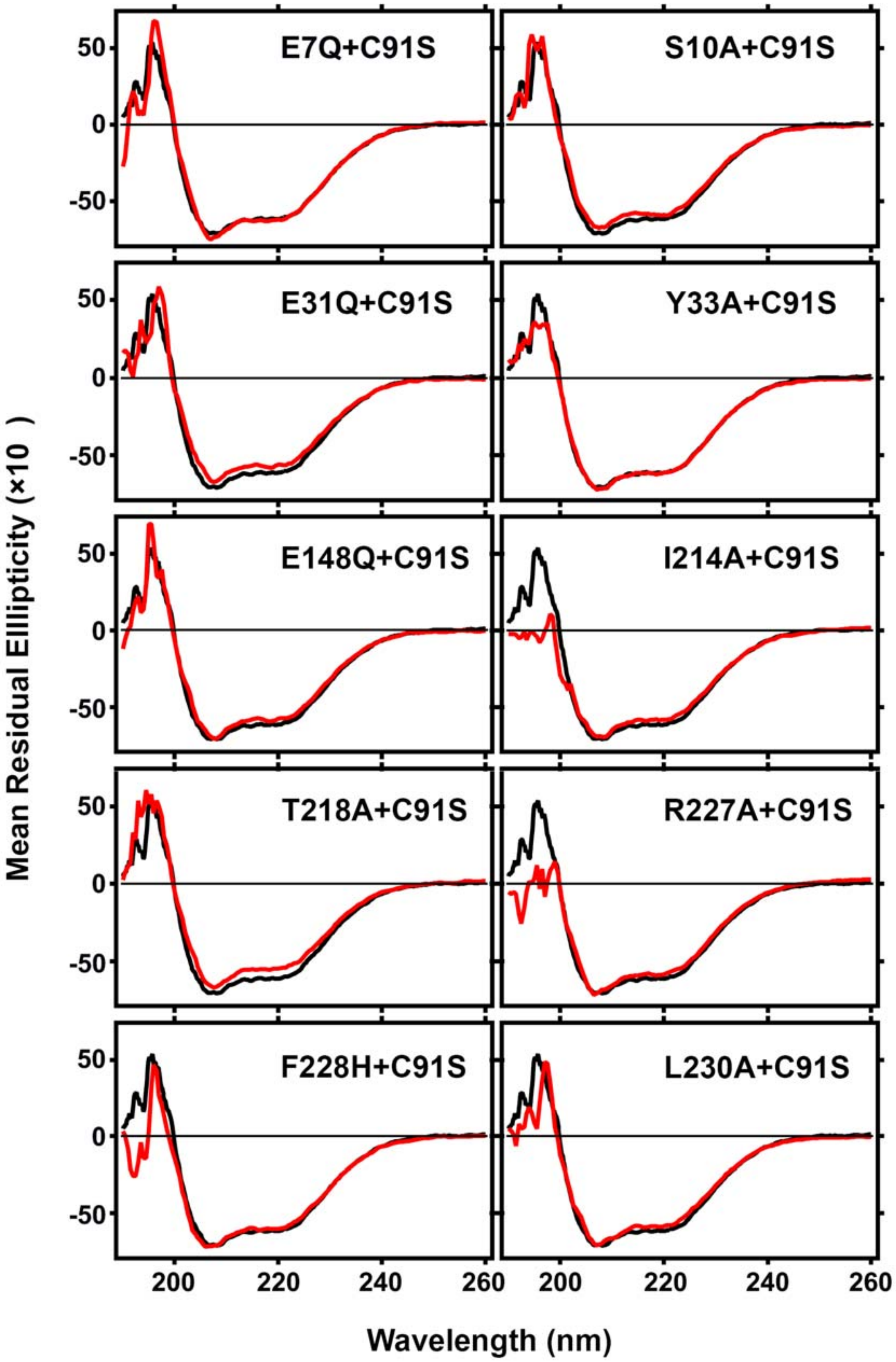

Supplementary Figure S5

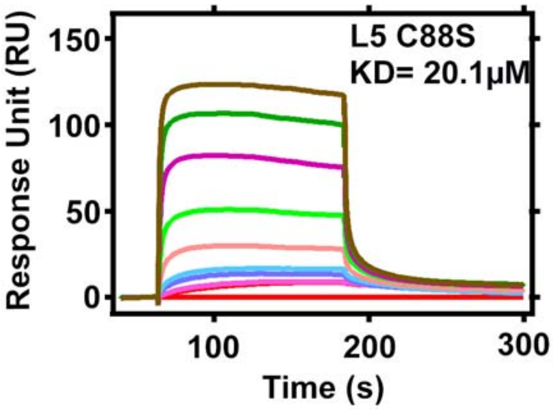

Supplementary Table S1. Rate of Ub-AMC Hydrolysis by wild-type BAP1N and its mutants.

Protein	Rate (pM/sec)	Fold Change
WT	105.2 ± 4	1
E7Q	26.1 ± 3.3	4
S10A	0.8 ± 3.3	132
E31Q	0.4 ± 2.4	263
Y33A	0.7 ± 1.1	150
E148Q	89.1 ± 1.2	1.2
I214	16.2 ± 0.4	6.5
T218A	112.1 ± 4.7	1
R227A	10.5 ± 3.5	10
F228H	11.7 ± 0.2	9
L230A	45.1 ± 6.1	2.3

Supplementary Table S2. Secondary structure analysis of BAP1N single mutants

BAP1 Double Mutants	Helix Percentage	Strand Percentage	Turn Percentage	Unordered Percentage
WT	20	25	25	30
E7Q	21	22	17	40
S10A	21	24	26	30
E31Q	22	18	16	44
Y33A	18	24	21	37
E148Q	20	21	17	42
I214A	19	23	16	41
T218A	18	25	20	37
R227A	19	24	14	43
F228H	20	24	26	31
L230A	20	19	16	45

Supplementary Table S3. Thermal melting point of wild-type BAP1N and its mutants.

**Enzyme**	**Tm**	**Std. Dev.**
WT	47.9	0.1
E7Q	49.3	1.1
S10A	50.8	0.1
E31Q	49.4	0.2
Y33A	49.3	0.3
E148Q	49.2	0.2
I214A	45.2	0.1
T218A	49.4	0.2
R227A	44.6	0.02
F228H	48.9	0.2
L230A	42.9	0.2

Supplementary Table S4. SPR-derived binding constants for the interaction between different BAP1N double mutants and immobilized ubiquitin.

**BAP1 Double Mutants**	**K_D (μM)**	**Fold Change**
C91S	4 ± 1.6	1
E7Q	5.6 ± 1.4	1
S10A	82.4 ± 7.1	21
E31Q	108.8 ± 4.5	27
Y33A	51.6 ± 4.5	13
E148Q	8.8 ± 2.5	2
I214A	45 ± 3.5	11
T218A	16.9 ± 1.4	4
R227A	53.2 ± 8.2	13
F228H	33.8 ± 5.9	9
L230A	23.2 ± 3.2	6
